# High Temperature can Change Root System Architecture and Intensify Root Interactions of Plant Seedlings

**DOI:** 10.3389/fpls.2020.00160

**Published:** 2020-02-26

**Authors:** Hongxia Luo, Han Xu, Chengjin Chu, Fangliang He, Suqin Fang

**Affiliations:** ^1^ School of Life Sciences, Sun Yat-sen University, Guangzhou, China; ^2^ Research Institute of Tropical Forestry, Chinese Academy of Forestry, Guangzhou, China; ^3^ Department of Renewable Resources, University of Alberta, Edmonton, AB, Canada

**Keywords:** root system architecture, root interaction, temperature change, root growth dynamics, root depth and width, species competition

## Abstract

Climate change could alter plant aboveground and belowground resource allocation. Compared with shoots, we know much less about how roots, especially root system architecture (RSA) and their interactions, may respond to temperature changes. Such responses could have great influence on species'acquisition of resources and their competition with neighbors. We used a gel-based transparent growth system to *in situ* observe the responses of RSA and root interactions of three common subtropical plant species seedlings in Asia differing in growth forms (herb, shrub, and tree) under a wide growth temperature range of 18–34°C, including low and supra-optimal temperatures. Results showed that the RSA, especially root depth and root width, of the three species varied significantly in response to increased temperature although the response of their aboveground shoot traits was very similar. Increased temperature was also observed to have little impact on shoot/root resource allocation pattern. The variations in RSA responses among species could lead to both the intensity and direction change of root interactions. Under high temperature, negative root interactions could be intensified and species with larger root size and fast early root expansion had competitive advantages. In summary, our findings indicate that greater root resilience play a key role in plant adapting to high temperature. The varied intensity and direction of root interactions suggest changed temperatures could alter plant competition. Seedlings with larger root size and fast early root expansion may better adapt to warmer climates.

## Introduction

Temperature is one of the most important variables that influence plant growth (Gray and Brady, 2016). According to the fifth assessment of the IPCC, the global mean air temperature is predicted to increase by 0.3–4.8°C by the end of this century (Collins et al., 2013; Pau et al., 2018). The rapid warming projected for the planet and the limited ability of plants to track climate changes mean that species' survivorship under climate change critically depends on their thermal adaptation ability (Zhu et al., 2012; Lambers, 2015; Urban, 2015). This aspect of adaptation has so far been extensively explored through examining the plant growth response to temperature changes (Teskey and Will, 1999). Variation of aboveground shoot traits, such as shoot height, leaf morphology, and phenology, have been the focus of concern and are observed to change with increasing temperature (Chung et al., 2013; Meineri et al., 2014). However, although the root system architecture (RSA) which towards deploying roots in the soil that optimizes the acquisition of water and nutrient has been thought to be able to minimize the negative impact of temperature changes (de Dorlodot et al., 2007), compared with shoot, few studies have investigated the sensitivity in RSA response to temperature changes, because of the difficulty in direct observation on underground growth (Aidoo et al., 2016).

RSA describes the spatial configuration of the root system in soil and is critical for plants to adapt to different environments (Zhu et al., 2011; Bardgett et al., 2014). In highly heterogeneous soil environments, RSA is considered more important than morphology for nutrients and water uptake (Fitter, 1987). It has been shown that RSA can facilitate plant adaption to water and nutrition deficit conditions (Bell and Sultan, 1999), resist to disease and insect pest infection (Roman-Aviles et al., 2004), and mediate intra- and interspecific competition pressure (Tosti and Thorup-Kristensen, 2010; Fang et al., 2013; Belter and Cahill, 2015). The variation in RSA resulting from global change will impact not only plant performance by affecting nutrition acquisition (de Dorlodot et al., 2007), but also competition under field conditions by changing belowground interactions (Caffaro et al., 2011).

RSA responses to increased temperature can be species-specific, as different species often have different optimum temperatures for root growth (Gray and Brady, 2016). Previous studies show the effect of increasing temperature on root growth of plant seedlings can be promotive (Domisch et al., 2001; Lahti et al., 2005), inhibitive (Forbes et al., 1997), or first promotive then inhibitive after an optimum temperature is reached (Seiler, 1998). Even for species sharing the same habitat, their RSA can have species-specific responses to increased temperature (Bardgett et al., 2014). The variations in RSA response to increased temperature among species can also change competition among plant communities (Weltzin et al., 2003).

A question of important significance to plant adaptation to increasing temperature is how plant adjust belowground and aboveground biomass allocation pattern and resource acquisition traits to better adapt to the change in climate (Aidoo et al., 2016). Analyzing the difference between shoot and root traits in response to temperature changes will help determine which process— carbon fixation or nutrition acquisition— limits plant growth under increased temperature (Lynch and St. Clair, 2004; Craine et al., 2005).

Different root responses to increased temperature may also result in different root interactions with intra- and interspecific plants. According to the stress-gradient hypothesis, adverse living conditions caused by temperature change could transform negative species interactions into positive species interactions (He et al., 2013). Temperature might not only affect the strength of root interactions but also change the direction. Positive plant interactions describe beneficial behaviors between plants, which widely exist in nature (Callaway, 1995). In extreme environments where positive plant interactions dominate, the presence of neighbors can improve the soil environment (Gold and Bliss, 1995) and provide nursery effect (Carlsson and Callaghan, 1991), thus enhance the performance of focal species. Under climate change, many studies have suggested that interaction, mainly competition, could be the primary driver for species composition and vegetation dynamics (Alexander et al., 2015; Ettinger and HilleRisLambers, 2017). However, these findings are mostly inferred from the aboveground growth of plants, leaving belowground processes unexplored. In some circumstances, the aboveground response could synergistically interact with belowground changes (Belter and Cahill, 2015).

Previous studies on how temperature affects plant roots mainly focus on traditional root growth metrics, such as root biomass and length for seedlings (Lahti et al., 2005), and fine-root morphology for mature trees (Valverde-Barrantes et al., 2017) by destructive sampling without *in situ* observation and measurement. Key RSA traits such as root depth and width are rarely studied, although they are important in resource competition. In this study, to precisely measure RSA traits, we grew plants in a newly invented three-dimensional (3D) transparent solid growth system, from which *in situ* RSA of plant seedlings can be dynamically observed (Fang et al., 2009).

This work addresses three questions: (1) Sensitivity of RSA traits— for inferring plant seedling adaptation to warming climate: Which RSA traits respond sensitively to temperature change and how they change with temperature? (2) Comparisons between root/shoot growth traits in response to increased temperature— for understanding how plant seedlings coordinate root/shoot responses to climate change: Do roots of plant seedlings respond more strongly than shoots to change in temperature? (3) Interspecific root interactions: How does an increase in temperature change the intensity and the direction of root interactions? To answer these questions, we studied RSA traits of three plant species seedlings with different life forms for understanding the responses of root traits to temperature change.

## Materials and Methods

### Plant Materials and Experimental Design

#### Plant Materials

Three common subtropical plant species with different RSA from Heishiding (HSD) Natural Reserve (N23.27°, E11.15°, Guangdong province, China) were used in our experiment: *Corchorus capsularis* L. (Tiliaceae), *Mimosa sepiaria* Benth. (Fabaceae, Mimosoideae), and *Ormosia glaberrima* Y. C. Wu (Fabaceae, Papilionoideae). They have different life forms. *C. capsularis* is an annual herb that prefers warm-humid climate and is native to tropical Asia. *M. sepiaria* is a shrub that usually grows in sunny habitats and originates from tropical America, but is commonly found in Guangdong province. *O. glaberrima* is a local evergreen tree species and fond of sunny habitats. The seeds used in our experiment were all collected from HSD Natural Reserve and we got the permission of the administrative departments of the nature reserves. Dr. Han Xu from Research Institute of Tropical Forestry, Chinese Academy of Forestry, China conducted the formal identification of the samples and the voucher specimens were deposited in South Campus of Sun Yat-sen University (N23.09°, E113.29°, Guangzhou city, Guangdong province, China).

#### Temperature Treatments

To examine plant performance under a wide range of temperature, we set five temperature levels: 18/13, 22/17, 26/21, 30/25, and 34/29°C (day/night), and the length of day and night was set to 12 h for all temperature regimes respectively based on our preliminary experiment. 30/25°C was assumed to be the optimum temperature for these three species according to the plant aboveground biomass in a preliminary experiment. This “optimum” temperature was set up according to the local observed mean day/night temperature (31.1/26.5°C) of the hottest month (July) in HSD Natural Reserve. 18/13 and 34/29°C were regarded as the low and supra-optimal temperatures, respectively.

#### Plant Growth

**Figure 1:**
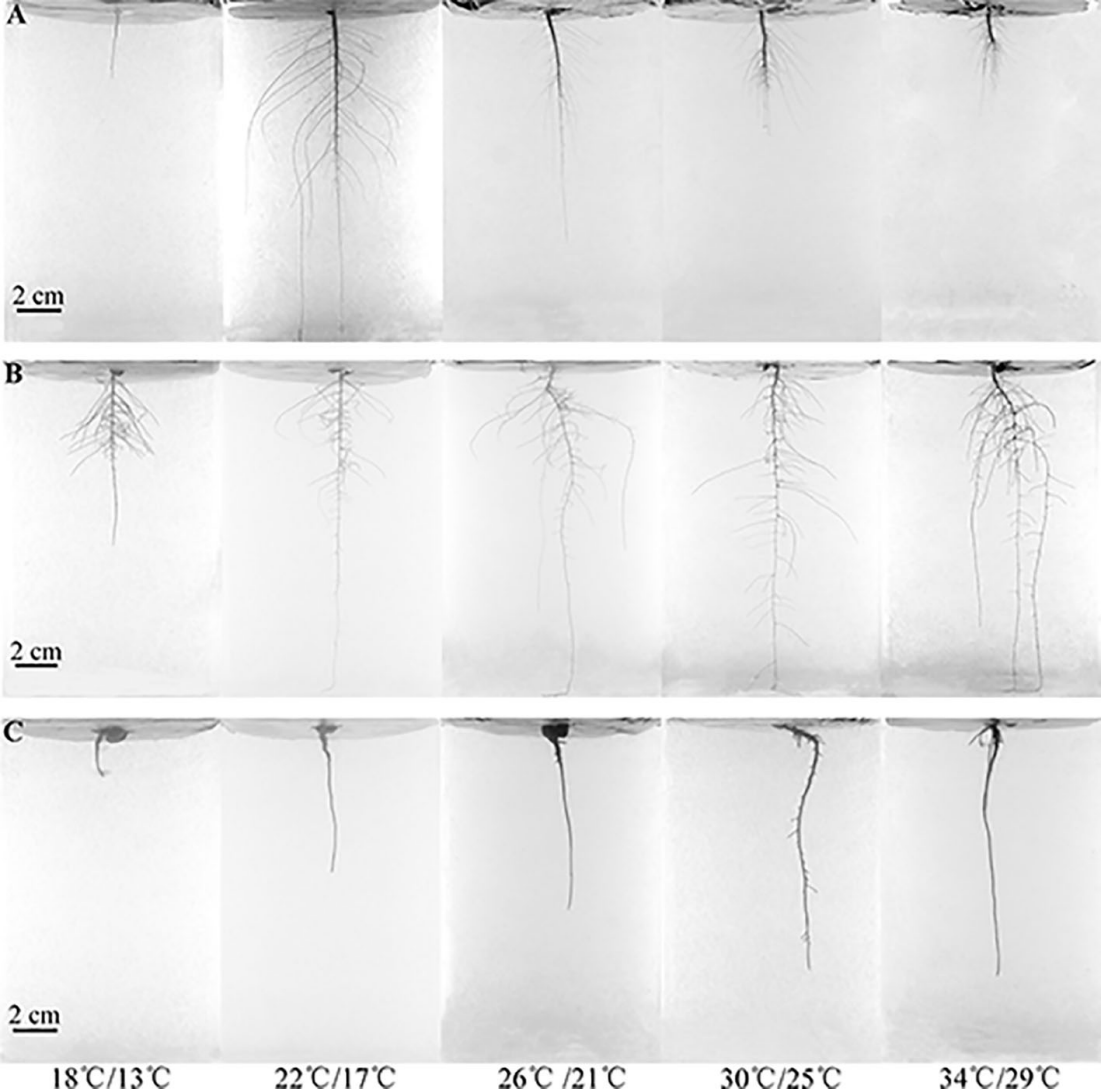
Root system architecture changes under different temperatures on the 84th day. **(A)** *C. capsularis*, **(B)** *M. sepiaria*, **(C)** *O. glaberrima*.

Besides monoculture growth of each of the three species (**Figure 1**), *C. capsularis* and *M. sepiaria* each was also grown in species-pair combinations, under the above five temperature levels to explore temperature effects on root interactions (**Supplementary Figure S1**). In total, there were five species-pair combinations (*C. capsularis-C. capsularis*, *M. sepiaria-M. sepiaria*, *C. capsularis-M. sepiaria*, *C. capsularis-O. glaberrima*, *M. sepiaria-O. glaberrima*). Since contamination in 3D transparent solid growth system could limit root visibility *in situ* and affect plant growth, we set each combination 6–12 independent replicates under each temperature treatment to make sure each combination at least has three independent biological replications at harvest without contamination.

The species-pair combination between *O. glaberrima-O. glaberrima* seedlings cannot be detected by RSA, because they give priority to the tap root growth and then there are not enough lateral roots developed, which lead to less competitive interactions. Therefore, we do not consider *O. glaberrima-O. glaberrima* here.

The *C. capsularis* seeds were surface-sterilized with 30% H_2_O_2_ for 4 h, and the *M. sepiaria* seeds sterilized with 20% H_2_O_2_ for 1 h. The *O. glaberrima* seeds were treated with concentrated H_2_SO_4_ for 30 min and rinsed with sterile water to destroy the outer hard layer of the seeds to promote germination, then sterilized with 15% H_2_O_2_ for 30 min. The seeds were then rinsed with sterile water and sown in petri dishes in the dark at 32°C to germinate.

The germinated seedlings were then transplanted to transparent cylinders, 20 cm in height and 10 cm in diameter, filled with 1.2 L transparent solid growth medium made from half-strength Hoagland solution and 0.2% Phytagel TM (Sigma-Aldrich, German, pH = 5.8) (**Figure 1**, **Supplementary Figure 1**). Differing from the soil or sand-based growth systems, ours allows 3D observation and measures of root growth while having similar RSA with those under soil condition (Clark et al., 2011). In the interaction experiment, the distance between the seeds of the two plants was 2 cm. Foil was used to cover the surface of the growth medium and wrap the containers to create a dark environment for root growth (Fang et al., 2011). After transplantation, the containers were moved to plant growth chambers of the same light and humidity conditions, except for temperature treatment.

#### Trait Measurement

All the plants were harvested 12 weeks (84 days) after germination before most of roots touching the cylinder bottom to avoid container effect (a few individuals of *M. sepiaria* were inevitably touch the bottom of the cylinder for their fast growth). Few contaminated replications were not used after harvest. In total, 117 individuals of *C. capsularis*, 141 individuals of *M. sepiaria,* and 24 individuals of *O. glaberrima* were used (details in sample size showed in **Supplementary Table 1**). Root analysis software, WinRHIZO (Pro 2013a, Regent Instrument Inc.), was used to measure the RSA parameters, including total root length, root surface area, root volume, average root diameter, and number of root tips. Root branching intensity was recorded as the ratio of number of root tips and total root length (Kramer-Walter et al., 2016). We also measured stem length. The aboveground, belowground, and leaf biomass was measured after drying in an oven at 75°C for 48 h. Most importantly, root width and depth and their growth dynamics were measured *in situ* through imaging every week from the 3rd day after germination, for a total of 13 root image sessions for each plant. The root width was recorded as the maximum horizontal distance of lateral roots. The root depth was the maximum vertical root length, including root length on the bottom of containers if the roots have already touched the bottom of the container. We also obtained leaf area (cm^2^) from the scanned images. The dynamic images of roots all were analyzed by Image J (Version 1.49, National Institutes of Health).

The seven RSA traits obtained broadly describe the entire root system responses to temperature. They play different physiological functions and can be roughly divided into four categories. (1) Nutrient/water uptake traits (total root length, surface area, and volume) which are correlated and directly reflect plant nutrient uptake and competition ability (Casper and Jackson, 1997). (2) Resource occupancy traits (root width and depth) which reflect plant horizontal and vertical soil resource occupancy ability, respectively (Belter and Cahill, 2015). (3) Nutrient transport traits (average root diameter) which are associated with multiple functions, including nutrient transport, soil penetration and anchorage (Kong et al., 2014). (4) Nutrient foraging traits (root branching intensity) which are critical for nutrient foraging and play an important role in the response of roots to nutrient patches (Kong et al., 2014; Kramer-Walter et al., 2016). Higher root branching intensity usually results in a thinner root diameter (Kaspar and Bland, 1992). The nutrition absorption and resource occupancy associated RSA traits both are size-related metric (Belter and Cahill, 2015).

### Statistical Analysis

#### Temperature Response Analysis

Tukey-Kramer HSD multiple comparison method (DTK packages) was used to make comparisons of trait parameters under different temperature treatments.

#### Root Interaction Analysis

The relative interaction intensities (RII, equation 1) (Armas et al., 2004) were calculated to describe the direction and intensity of root interactions and show how they change with temperature.

(1) $$\text{RII}=(V_{D}-V_{A})/(V_{A}+V_{D})$$

where *V_A_* is the trait value of plants growing alone, and *V_D_* is the trait value of focal species when growing with other species.

We used type III two-way ANOVA to examine whether the effects of root interactions on focal species growth (in the form of total biomass accumulated) were affected by neighbor identity, temperature, and their interaction. Then a one-sample *t*-test was used to examine the significance of root interactions.

#### RSA Dynamics Analysis

*O. glaberrima* usually has only one short tap root, sometimes with a few tiny, short lateral roots at the seedling stage. Thus, we modeled only root width and depth of *C. capsularis* and *M. sepiaria* as a function of plant age using the three-parameter asymptotic model proposed by Paine et al. (2012). The root width expansion of the two species was well fitted by a monomolecular model (equation 2), whereas root depth expansion was well fitted by the three-parameter logistic model (equation 3).

(2) $${\text{M}}_{\text{width}}=\text{K}-{\text{e}}^{-\text{rt}}(\text{K}-{\text{M}}_{0})$$

(3) $${\text{M}}_{\text{depth}}=\frac{{\text{M}}_{0}\text{K}}{{\text{M}}_{0}+(\text{K}-{\text{M}}_{0}){\text{e}}^{-\text{rt}}\;}$$

(4) $${\text{AER}}_{\text{width}}=\frac{{\text{dM}}_{\text{width}}}{\text{dt}}={\text{re}}^{-\text{rt}}(\text{K}-{\text{M}}_{0})$$

(5) $${\text{AER}}_{\text{depth}}=\frac{{\text{dM}}_{\text{depth}}}{\text{dt}}=\frac{{\text{rM}}_{0}{\text{Ke}}^{-\text{rt}}(\text{K}-{\text{M}}_{0})}{{\left({\text{M}}_{0}+{\text{e}}^{-\text{rt}}(\text{K}-{\text{M}}_{0})\right)}^{2}}$$

Where M is accumulated growth, M_0_ is initial size, K is asymptotic size, r is rate parameter, and t is time.

To compare and visualize the difference of root width and depth growth dynamics among temperature treatments, we compiled the root width and depth growth data of each species in monoculture and fitted root width and depth expansion grouped by temperature treatment and obtained the function-derived absolute expansion rate (AER) (equation 4 and 5), which was similar to the absolute growth rate (AGR) proposed by Paine et al. (2012).

All of the above analyses were conducted by R software (R Core Team, 2016).

## Results

### RSA Responses to Temperature Changes

**Figure 2:**
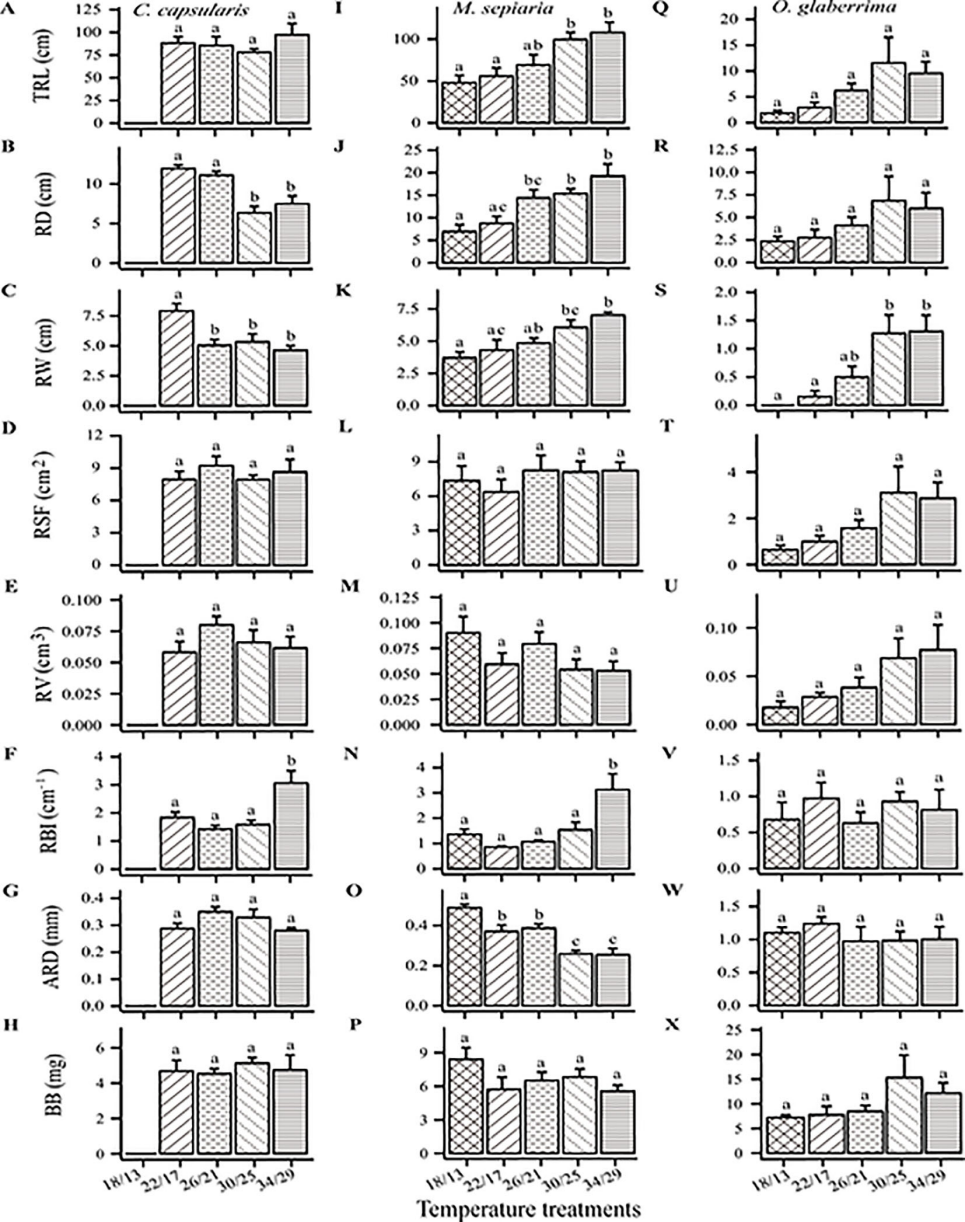
Temperature effects on seven root architecture traits and belowground biomass. TRL, total root length; RD, root depth; RW, root width; ARD, average root diameter; RBI, root branching intensity; RSF, root surface area; RV, root volume; BB, belowground biomass. **(A–H)** *C. capsularis*, **(I–P)** *M. sepiaria*, **(Q–X)** *O. glaberrima.* Error bars represent standard error (±SE). Different letters denote significant level at *P* < 0.05.

The three species differed in RSA responses to increased temperature from low (18°C day/13°C night) to high temperature (34/29°C) (**Figures 1** and **2**). At 18/13°C, *C. capsularis* plants were all dead while the other two species survived. Interestingly, at 22/17°C, only 4°C above the fatal low temperature, the root depth and width of *C. capsularis* reached the largest value, which was significantly larger than that at 30/25°C and 34/29°C, whereas there were no significant differences in *C. capsularis* total root length, surface area and volume among the four temperature treatments (**Figures 1A** and **2A–E**). For *M. sepiaria*, total root length, root depth, and root width all increased with increasing temperature (**Figures 1B** and **2I–K**), but there were no significant temperature effects on root surface area and root volume (**Figures 2L, M**). *O. glaberrima* seedlings usually have only the tap root, sometimes with a few tiny, short lateral roots (**Figure 1C**). Except for root width, no significant responses of other RSA traits triggered by temperature changes were detected for *O. glaberrima* (**Figures 1C** and **2Q–X**).

In addition to the above root size-associated RSA traits, high temperature (34/29°C) significantly affected the nutrient foraging and transport-associated RSA traits of *M. sepiaria* (**Figures 2N, O**), but only transport-associated RSA traits of *C. capsularis* (**Figure 2F**). The high temperature significantly increased the branching intensity of *C. capsularis* (**Figure 2F**) and *M. sepiaria* (**Figure 2N**), and decreased the average root diameter of *M. sepiaria* (**Figure 2O**).

### RSA Dynamics Responses to Temperature Changes

**Figure 3:**
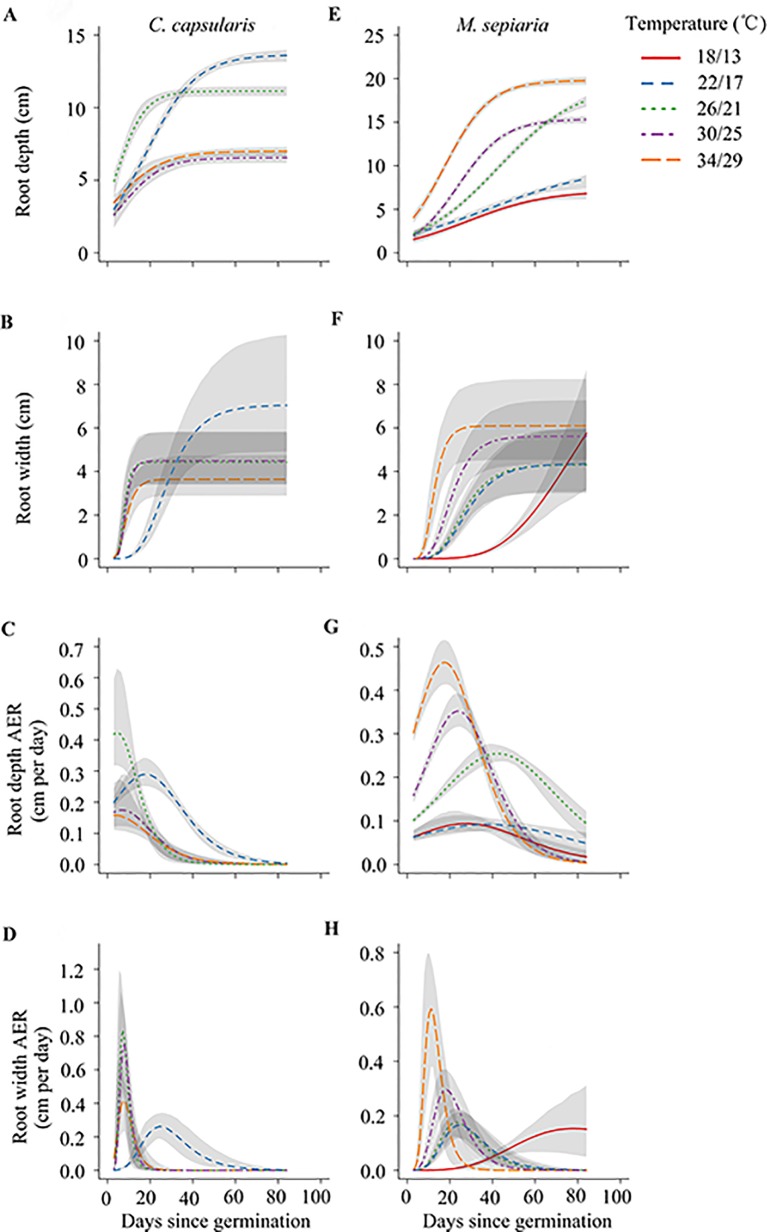
Temperature effects on the dynamics of two root size-related metrics and their absolute expansion rate (AER) of two species under different temperature treatments. Graphs show predicted values with 95% confidence intervals (gray curves) for **(A**–**D)** *C. capsularis*, **(E**–**H)** *M. sepiaria*.

During the whole experimental period, the root depth and width expansion of *C. capsularis* and *M. sepiaria* first gradually increased with time and then reached an asymptote with no further expansion. However, among temperature treatments, the steepness (which is quantified by the duration of the expansion-increasing phase) and asymptotic value of root expansion were different. Those differences can be best explained by changes in absolute expansion rate (AER) over time (**Figure 3**).

For *C. capsularis*, the peak AER of root depth and width first increased with temperature and then decreased after reaching the optimum temperature. Both root depth and width of *C. capsularis* had an optimum temperature around 26/21°C for AER. At 26/21°C, the timing to reach peak AER was nearly the shortest (**Figures 3C, D**; **Supplementary Table 2**). For *M. sepiaria*, the peak AER of root depth and width increased with increasing temperature (**Figures 3G, H**; **Supplementary Table 3**). The timing to peak root width AER was shortened with increasing temperature (**Figure 3H**; **Supplementary Table 3**). Inconsistent with root width, the timing to peak root depth AER was first extended and then shortened after reaching its maximum at 26/21°C (**Figure 3G**; **Supplementary Table 3**).

### Shoot Traits and Shoot/Root Ratio Responses to Temperature Change

**Figure 4:**
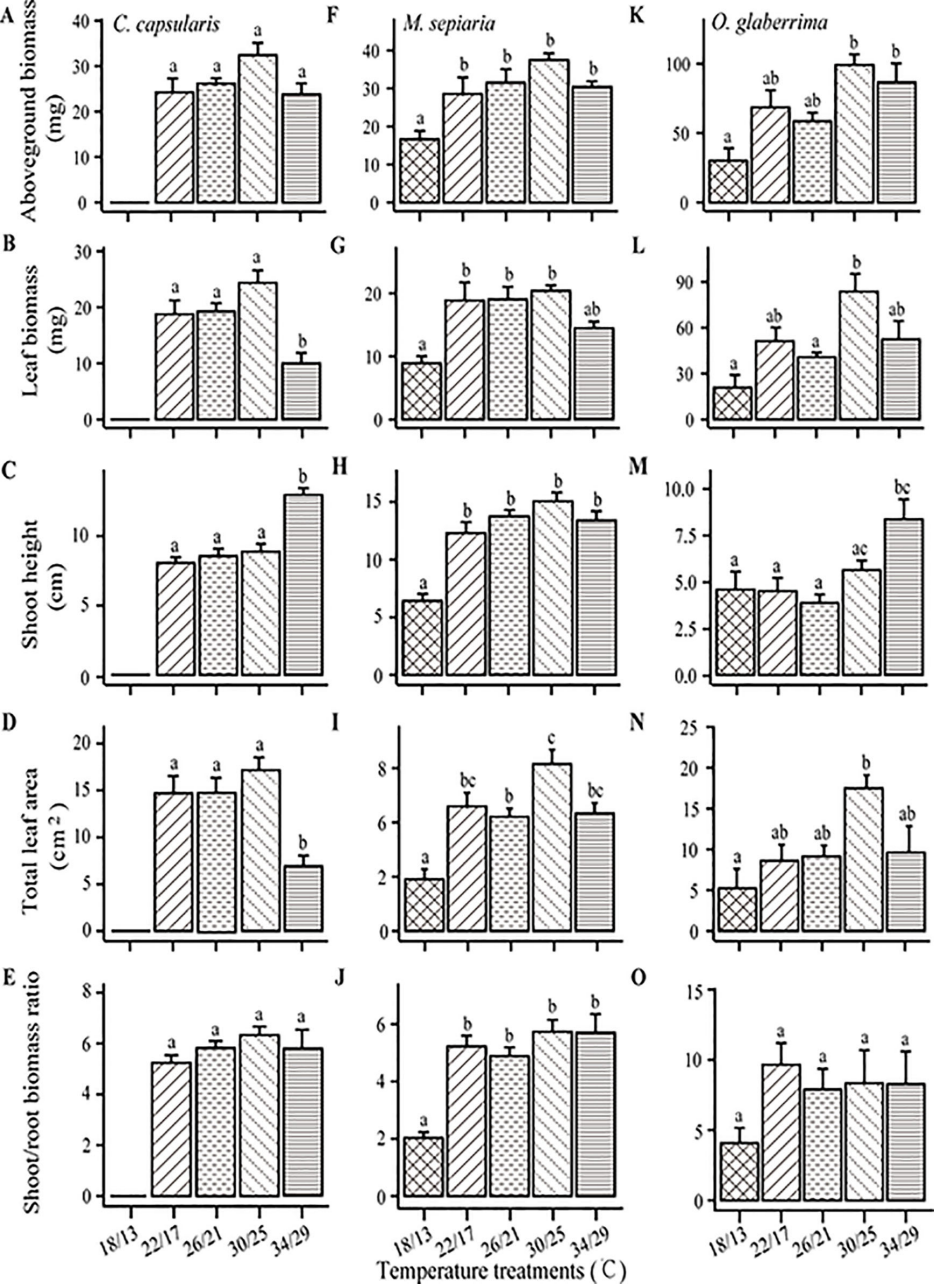
Temperature effects on shoot traits and the ratio of shoot biomass to root biomass. **(A**–**E)** *C. capsularis*, **(F**–**J)** *M. sepiaria*, **(K**–**O)** *O. glaberrima*. Error bars represent standard error (±SE). Different bar annotations denote significant differences (*P* < 0.05).

Shoot traits of the three species showed high sensitivity to temperature changes (**Figure 4**). Although shoot trait responses to temperature change differed in their details, the response trends were similar. All the three species had an optimum temperature for aboveground biomass at 30/26°C (**Figures 4A, F, K**), a decrease in leaf biomass and area at 34/29°C (**Figures 4B, G, L, D, I, N**), an increasing trend for stem length with increasing temperature (**Figures 4C, H, M**). Furthermore, there was no significant change in shoot/root biomass ratio of the three species when increasing temperature from 22/17 to 34/29°C (**Figures 4E, J, O**). Low temperature (18/13°C) significantly decreased shoot/root biomass ratio of *M. sepiaria* (**Figure 4J**).

### Root Interaction (Intensity and Direction) Responses to Temperature

Results of type III two-way ANOVA analysis showed that for *M. sepiaria* as the focal species, the effect of root interaction on its growth was significantly influenced by temperature (*P* < 0.0001), neighbor identities (*P* = 0.0343), and the interaction between temperature and neighbor identities (*P* = 0.0221). However, for *C. capsularis* as the focal species, the root interaction effect on its growth was significantly influenced by temperature (*P* = 0.0002) and the interaction between temperature and neighbor identities (*P* < 0.0001), but not neighbor identities (*P* = 0.310) (**Table 1**).

**Table 1:** ANOVA for the effects of root interactions on focal species growth.

Parameters	*C. capsularis*				*M. sepiaria*			
	Sum Sq	Df	*F* value	*Pr*(>*F*)	Sum Sq	Df	*F* value	*Pr*(>*F*)
Intercept	0.387	1	22.64	<0.0001	0.0584	1	4.27	0.0416
Temperature	0.363	3	7.09	0.0002	0.448	4	8.17	<0.0001
Neighbors	0.0406	2	1.19	0.310	0.0957	2	3.49	0.0343
Temperature× Neighbors	0.754	6	7.36	<0.0001	0.261	8	2.38	0.0221
Residuals	1.38	81			1.32	96		

Plant total biomass is used to represent focal species growth. The effects of root interactions on focal species are assumed to be affected by neighbor identity and temperature.

**Figure 5:**
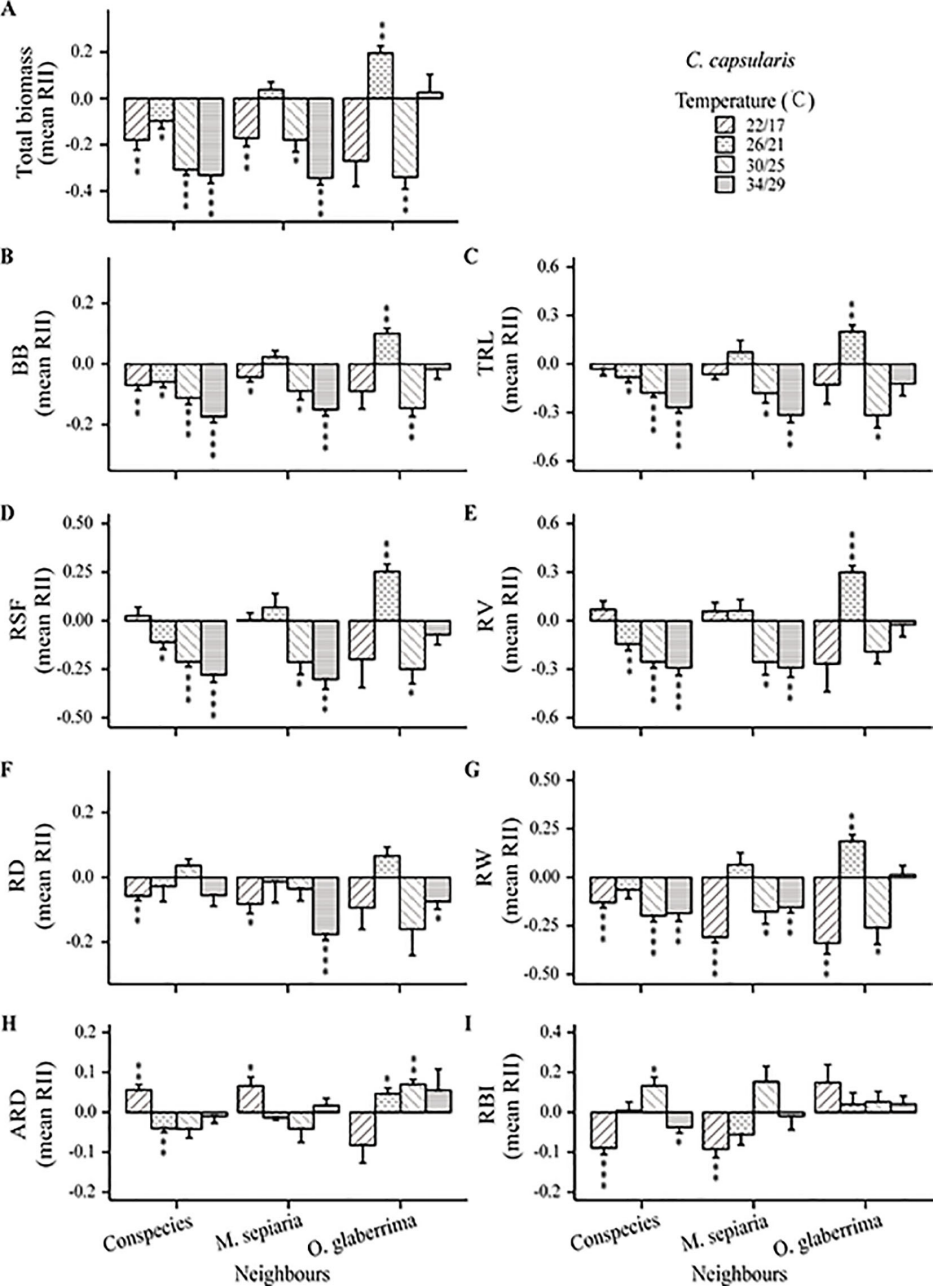
Temperature effects on root relative interaction intensity (RII) with focal species *C. capsularis*. Graphs show RII measured by **(A)** Total biomass, **(B)** BB, belowground biomass, **(C)** TRL, total root length, **(D)** RSF, root surface area, **(E)** RV, root volume, **(F)** RD, root depth, **(G)** RW, root width, **(H)** ARD, average root diameter and **(I)** RBI, root branching intensity. The bars are grouped by species-pair combination treatments and different bars represent different temperature treatments. Asterisks indicate one-sample t-tests for the difference from zero. *, P < 0.05; **, P < 0.01; ***, P < 0.001.

**Figure 6:**
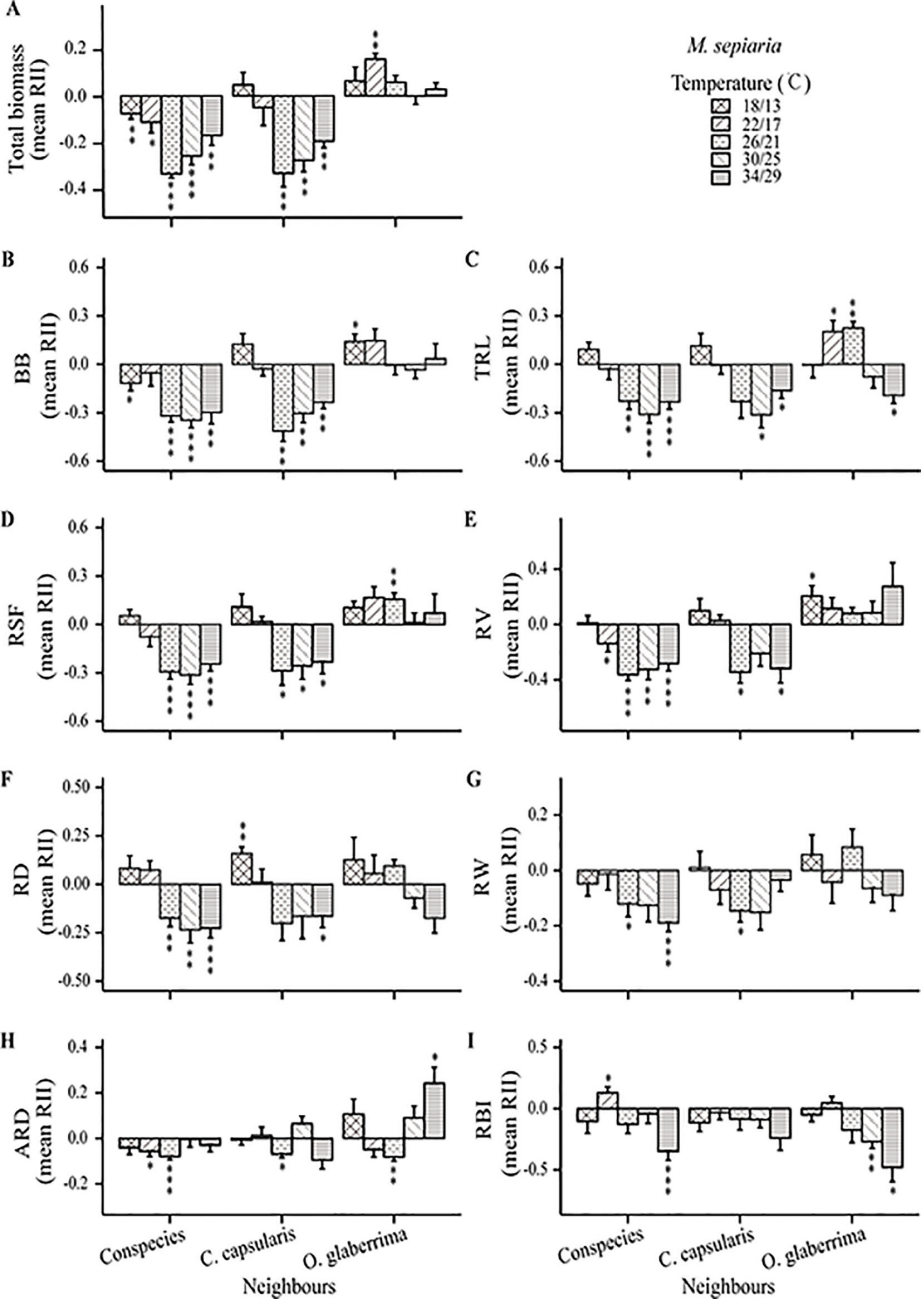
Temperature effects on root relative interaction intensity (RII) with focal species *M. sepiaria*. Graphs show RII measured by **(A)** Total biomass, **(B)** BB, belowground biomass, **(C)** TRL, total root length, **(D)** RSF, root surface area, **(E)** RV, root volume, **(F)** RD, root depth, **(G)** RW, root width, **(H)** ARD, average root diameter and **(I)** RBI, root branching intensity. The bars are grouped by species-pair combination treatments and different bars represent different temperature treatments. Asterisks indicate one-sample t-tests for the difference from zero. *, P < 0.05; **, P < 0.01; ***, P < 0.001..

The intensity and direction of root interactions were affected by temperature change for both *C. capsularis* and *M. sepiaria* as the focal species (**Figures 5** and **6**). The growth of *C. capsularis* and *M. sepiaria* was significantly inhibited when grown with conspecies under all the temperature treatments with different intensities (**Figures 5A** and **6A**). Growth promotion was mainly observed in mixture with other species under some temperature treatments. Significant total growth in *C. capsularis* was accompanied by significant increase in root biomass, total length, surface area, volume, width, and average diameter (**Figure 5**), whereas only total root length accompanied significant growth in *M. sepiaria* (**Figure 6**). For *C. capsularis*, growing with *O. glaberrima* significantly facilitated its growth at 26/21°C (**Figure 5A**). For *M. sepiaria*, growing with *O. glaberrima* facilitated its growth under all the temperature treatments except for a weak growth inhibition at 30/25°C, whereas a significant increase was only observed at 22/17°C (**Figure 6A**). However, we did not observe root interactions significantly promoted plant growth at 18/13, 22/17, or 34/29°C. Conversely, the greatest growth inhibition of *C. capsularis* was observed when it was planted with *M. sepiaria* at 34/29°C (**Figure 5A**).

## Discussion

The key finding of this study is that RSA parameters, especially root width and root depth, are highly sensitive to temperature change. Species from the same community may have adapted to similar habitats, but they do not show a consistent and directional response in RSA to temperature changes. However, compared with RSA, responses of shoots are conserved across the three species. The variations in RSA responses among species also increased the intensity and changed direction of root interactions in response to temperature changes. Although our experiments were conducted in very artificial conditions, a previous study conducted by Clark et al. (2011) have showed plants in gel having similar RSA with those under soil condition. These results thus are important to understand the complicated effect of increasing temperature on the performance and competition of plants at the early/establishment stage in natural environment.

Our results show that the three species vary in their RSA responses to temperature changes, especially root depth and root width. *C. capsularis* had a larger root system under low temperature, whereas the size of *M. sepiaria* roots generally increased with increasing temperature and *O. glaberrima* had the largest root size around the optimum temperature (**Figures 1** and **2B–C, I–K, Q–S**). A larger root system could help acquire more soil resources and increase nutrient uptake (de Kroon et al., 2003). The differences in root size of *C. capsularis* and *M. sepiaria* to temperature change suggest that *C. capsularis* may adapt better to relative low temperatures, whereas *M. sepiaria* may be better adapted to high temperatures. Although total root length can also be a measure of root size, increase in total root length but no changes in root depth and width can result in self-competition and reduce nutrient absorption efficiency (Kaspar and Bland, 1992). Besides, increased temperature was usually accompanied by decreases in water availability (Nord and Lynch, 2009), and deep root systems could ameliorate water deficit by increasing plant water uptake under warmer weather (Mueller et al., 2013). *M. sepiaria* might be better adapted to water deficit which comes after higher temperature with deeper roots.

Root branching intensity and average root diameter also exhibited high sensitivity to temperature change. High temperature significantly increased root branching of *C. capsularis* and *M. sepiaria* (**Figures 2F, N**), which is consistent with previous reports in crops (Nagel et al., 2009). This phenomenon might be caused by high temperature significantly accelerating the root meristem cell division, thus the development of lateral root primordium (Francis and Barlow, 1988). Kaspar and Bland (1992) reported that high root branching intensity usually results in thin root diameter. However, for *C. capsularis*, no significant change in the average root diameter was observed following root branching increase under elevated temperature (**Figures 2F, G**). Furthermore, warming experiments conducted on a temperate species, *Lactuca sativa*, suggested that increasing average root diameter under high temperature inhibited root nutrient acquisition under high temperature (Qin et al., 2007). In this study, decrease in the average root diameter of *M. sepiaria* under high temperature may improve root nutrient acquisition, suggesting that *M. sepiaria* is a better warm-adapted species (**Figure 2O**).

The responses of the root traits to temperature changes are also significantly different from shoot traits, reflecting the different effects of temperature on the aboveground and the belowground growth and resource allocation (Skarpaas et al., 2016). Root systems have narrow optimal growth temperature ranges, being extremely sensitive to environmental change and weak in adapting to harsh environments (Aidoo et al., 2016). In our experiment, although changes in temperature seemed to have no significant effect on root biomass of *C. capsularis* or *M. sepiaria* (**Figure 2**), RSA exhibited high sensitivity to temperature stress. For all the three species studied here, high temperature showed to increase stem length, resulting from the high demand for light (Meineri et al., 2014; Skarpaas et al., 2016), but reduced leaf area, because high temperature can increase photosynthetic rate which make leaves less important (Skarpaas et al., 2016). Meanwhile, increasing temperature had little impact on shoot/root resource allocation pattern (**Figure 4**). Plants usually allocate more resources to the organs that suffer more selection pressures and constraints (Husáková et al., 2018). The carbon fixation and nutrient acquisition can both limit plant growth under increased temperature. Moreover, our study found that the root expansion dynamics of *C. capsularis* and *M. sepiaria* had an asymptotic value for expansion and unimodal AER curve (**Figure 3**). These phenomena were also found in aboveground plant growth (Paine et al., 2012), which may be a growth strategy that plants take to respond to temperature change by balancing shoot and root growth or resource acquisition (Willaume and Pages, 2006).

Furthermore, the effectiveness of root response to competing neighbors depends not only on its extent but also its rapidity in response (Fitter, 1994; Bell and Sultan, 1999). In our experiments, the growth-promoting effect on *C. capsularis* at 26°C/21°C when interacting with *M. sepiaria* and *O. glaberrima* might result from its optimal root growth with large root sizes and earlier and faster root expansion (**Figures 1A**, **2B–C**, **3A–D** and **5A**; **Supplementary Table 2**). He et al. (2013) suggested that adverse living conditions caused by climate change might force negative species interactions into positive species interactions. Interestingly, at the supra-optimal temperature, we found only a weak growth-promoting effect on *C. capsularis* when interacting with *O. glaberrima,* but an intensified growth-inhibiting effect when interacting with *M. sepiaria* (**Figure 5A**). This result may be caused by the larger root system with earlier and faster expansion of *M. sepiaria* roots at the supraoptimal temperature (**Figures 1B**, **2I–K**, and **3E–H**; **Supplementary Table 3**). Our results further support the idea that species with a warmer thermal niche will increase in abundance under a warmer climate (Elmendorf et al., 2015), suggesting that species with greater root resilience have a competitive advantage.

## Conclusions

At the early/establishment stage, plants usually give priority to the root growth for nutrient uptake and physical support, especially in tropical regions for the intense competition (Radville et al., 2016). In summary, our study showed understanding the effect of warming on root systems of plant seedlings, especially the spatial distribution of root systems, is necessary to predict plant seedling performance and community regeneration in future climate warming. Our findings suggest that the RSA of the three plant seedlings from the same habitat show inconsistent responses to temperature changes. The variation of belowground responses to temperature change can be greater than the responses of the aboveground among species, suggesting that the differences in plant seedling adaptability to increased temperature are likely, at least partly, determined by belowground adaptive responses. Furthermore, the variation can also be an explanation for the direction and intensity of the change in plant interaction under climate warming, which also important for the success of seedling establishment. We suggest future studies should move on to field studies and compare the differences in RSA between artificial and natural conditions, and study the allocation of nutrients, such as nitrogen and phosphorus, between roots and shoots under climate warming, for better understanding how plants coordinate root/shoot growth and getting insight in tree seedling nurseries under climate change.

## Data Availability Statement

All data generated or analyzed during this study will be freely available upon request to corresponding author: SF (E-mail: fangsuq5@mail.sysu.edu.cn) for reasonable use only.

## Author Contributions

SF, HL, and FH designed the study. HL collected the data and performed the analysis. HL, SF, and HX led the writing. CC contributed to the revision. All authors contributed to discussion and writing. All authors read and approved the final manuscript.

## Funding

This work was funded by Pearl River S&T Nova Program of Guangzhou to SF (201610010082) and the National Natural Science Foundation of China Grant to SF (31370441).

## Conflict of Interest

The authors declare that the research was conducted in the absence of any commercial or financial relationships that could be construed as a potential conflict of interest.
