# Supplementary material for: High Temperature can Change Root System Architecture and Intensify Root Interactions of Plant Seedlings
Source: Front Plant Sci. 2020 Feb 26;11:160. doi: 10.3389/fpls.2020.00160 (PMC7054236; doi:10.3389/fpls.2020.00160)
Supplement: Supplementary file 1 [file DataSheet_1.docx]

Supplementary Material

# Supplementary Tables and Figures

## Supplementary Tables

**Supplementary Table 1.**  Sample size of *C. capsularis*, *M. sepiaria* and *O. glaberrima* harvested 12 weeks (84 days) after germination.

| Treatments | | Sample size |
| --- | --- | --- |
| *C. capsularis* grown alone | 22/17℃ | 6 |
|  | 26/21℃ | 6 |
|  | 30/25℃ | 5 |
|  | 34/29℃ | 7 |
| *M. sepiaria* grown alone | 18/13℃ | 6 |
|  | 22/17℃ | 6 |
|  | 26/21℃ | 6 |
|  | 30/25℃ | 7 |
|  | 34/29℃ | 5 |
| *O. glaberrima* grown alone | 18/13℃ | 3 |
|  | 22/17℃ | 6 |
|  | 26/21℃ | 6 |
|  | 30/25℃ | 4 |
|  | 34/29℃ | 5 |
| *C. capsularis*–*C. capsularis* | 22/17℃ | 12 |
|  | 26/21℃ | 9 |
|  | 30/25℃ | 12 |
|  | 34/29℃ | 10 |
| *M. sepiaria*–*M. sepiaria* | 18/13℃ | 10 |
|  | 22/17℃ | 10 |
|  | 26/21℃ | 10 |
|  | 30/25℃ | 10 |
|  | 34/29℃ | 11 |
| *C. capsularis* in *C. capsularis*–*M. sepiaria* pairs | 22/17℃ | 8 |
|  | 26/21℃ | 6 |
|  | 30/25℃ | 7 |
|  | 34/29℃ | 6 |
| *M. sepiaria* in *C. capsularis*–*M. sepiaria* pairs | 18/13℃ | 8 |
|  | 22/17℃ | 7 |
|  | 26/21℃ | 5 |
|  | 30/25℃ | 6 |
|  | 34/29℃ | 6 |
| *C. capsularis*  in *C. capsularis*–*O. glaberrima* pairs | 22/17℃ | 6 |
|  | 26/21℃ | 6 |
|  | 30/25℃ | 5 |
|  | 34/29℃ | 6 |
| *M. sepiaria* in *M. sepiaria*–*O. glaberrima* pairs | 18/13℃ | 5 |
|  | 22/17℃ | 6 |
|  | 26/21℃ | 6 |
|  | 30/25℃ | 6 |
|  | 34/29℃ | 5 |

**Supplementary Table 2.** The timing and magnitude of peak absolute expansion rates for root depth and width expansion of *C. capsularis.*

| Temperature treatments | *C. capsularis* | | | | |
| --- | --- | --- | --- | --- | --- |
|  | Root depth | |  | Root width | |
|  | Times/days | peak AER |  | Times/days | Peak AER |
| 22/17°C | 18 | 0.290 (0.251,0.348) | | 25 | 0.261 (0.184,0.340) |
| 26/21°C | 5 | 0.423 (0.321,0.603) | | 7 | 0.836 (0.502,1.09) |
| 30/25°C | 7 | 0.174 (0.124,0.299) | | 8 | 0.749 (0.476,0.944) |
| 34/29°C | 3 | 0.158 (0.112,0.265) | | 8 | 0.416 (0.231,0.550) |

**Supplementary Table 3.** The timing and magnitude of peak absolute expansion rates for root depth and width expansion of *M. sepiaria.*

| Temperature treatments | *M. sepiaria* | | | | | | |
| --- | --- | --- | --- | --- | --- | --- | --- |
|  | Root depth | |  | Root width | | | |
|  | Times/days | peak AER |  | Times/days | | | Peak AER |
| 18/13°C | 28 | 0.0940 (0.0768,0.120) | | | 78 | 0.154 (0.0543,0.292) | |
| 22/17°C | 38 | 0.0922 (0.0767,0.109) | | | 24 | 0.167 (0.119,0.214) | |
| 26/21°C | 42 | 0.255 (0.236,0.276) | | | 23 | 0.176 (0.128,0.224) | |
| 30/25°C | 23 | 0.352 (0.316,0.394) | | | 19 | 0.296 (0.218,0.380) | |
| 34/29°C | 23 | 0.352 (0.316,0.394) | | | 19 | 0.296 (0.218,0.380) | |

## Supplementary Figures


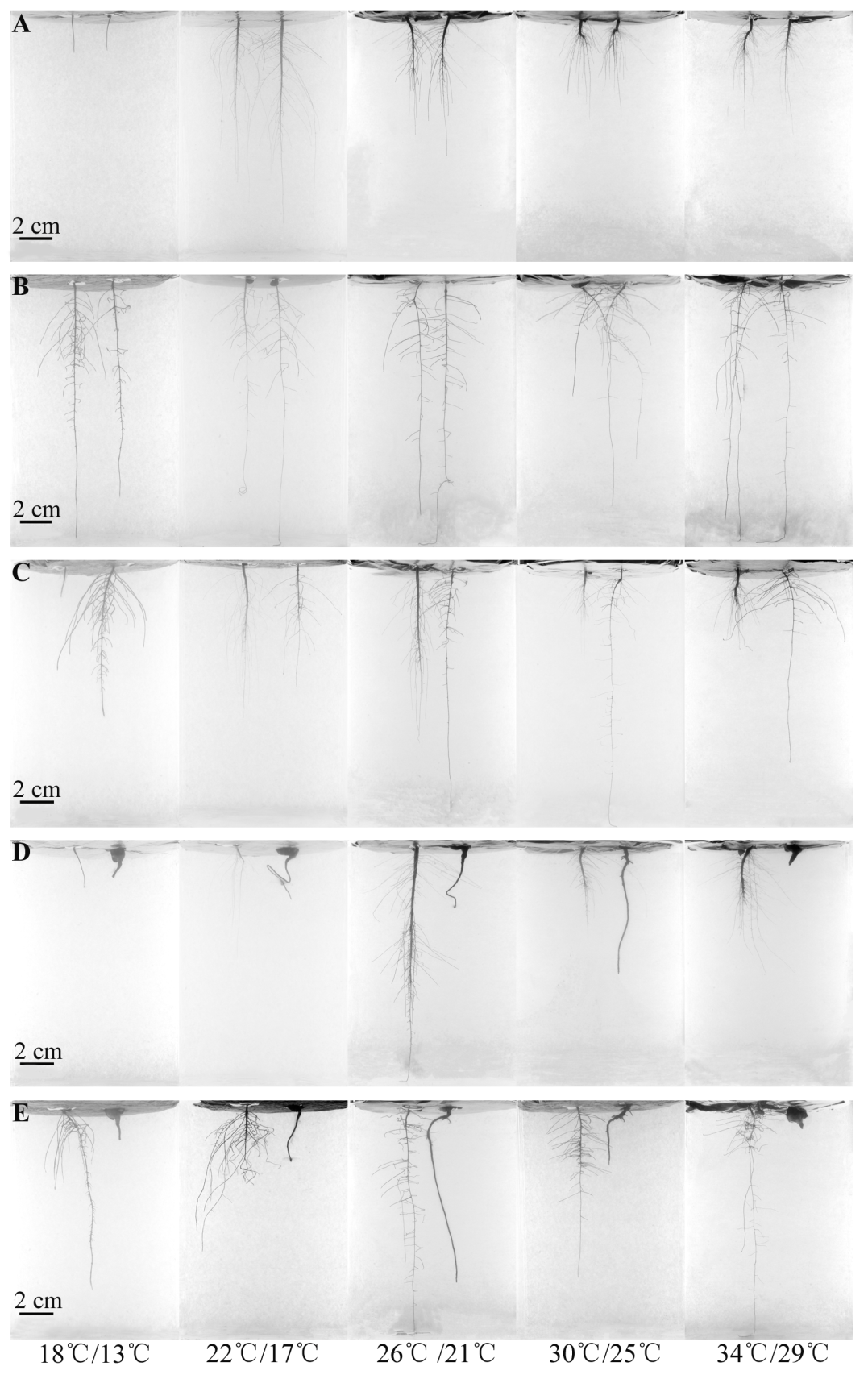


**Supplementary Figure 1.** RSA responses to interaction treatments under different temperatures. (A) *C. capsularis* interacting with conspecies, (B) *M. sepiaria* interacting with conspecies, (C) *C. capsularis* (left) interacting with *M. sepiaria* (right), (D) *C. capsularis* (left) interacting with *O. glaberrima* (right), (E) *M. sepiaria* (left) interacting with *O. glaberrima* (right).
